# Simple flow cytometric detection of haemozoin containing leukocytes and erythrocytes for research on diagnosis, immunology and drug sensitivity testing

**DOI:** 10.1186/1475-2875-10-74

**Published:** 2011-03-31

**Authors:** Rosangela Frita, Maria Rebelo, Ana Pamplona, Ana M Vigario, Maria M Mota, Martin P Grobusch, Thomas Hänscheid

**Affiliations:** 1 Instituto de Medicina Molecular, Faculdade de Medicina de Lisboa, Lisbon, Hospital Universitário de Santa Maria, Av. Prof. Egas Moniz, P-1649-028 Lisboa, Portugal; 2 University of Madeira, Funchal, Portugal; 3 Infectious Diseases, Tropical Medicine and AIDS, Division of Internal Medicine, Academic Medical Centre, University of Amsterdam, The Netherlands; 4 Institute of Tropical Medicine, University of Tübingen, Germany; 5 Medical Research Unit, Hôpital Albert Schweitzer, Lambaréné, Gabon

## Abstract

**Background:**

Malaria pigment (haemozoin, Hz) has been the focus of diverse research efforts. However, identification of Hz-containing leukocytes or parasitized erythrocytes is usually based on microscopy, with inherent limitations. Flow cytometric detection of depolarized Side-Scatter is more accurate and its adaptation to common bench top flow cytometers might allow several applications. These can range from the *ex-vivo *and *in-vitro *detection and functional analysis of Hz-containing leukocytes to the detection of parasitized Red-Blood-Cells (pRBCs) to assess antimalarial activity.

**Methods:**

A standard benchtop flow cytometer was adapted to detect depolarized Side-Scatter. Synthetic and *Plasmodium falciparum *Hz were incubated with whole blood and PBMCs to detect Hz-containing leukocytes and CD16 expression on monocytes. C5BL/6 mice were infected with *Plasmodium berghei *ANKA or *P. berghei *NK65 and Hz-containing leukocytes were analysed using CD11b and Gr1 expression. Parasitized RBC from infected mice were identified using anti-Ter119 and SYBR green I and were analysed for depolarized Side Scatter. A highly depolarizing RBC population was monitored in an *in-vitro *culture incubated with chloroquine or quinine.

**Results:**

A flow cytometer can be easily adapted to detect depolarized Side-Scatter and thus, intracellular Hz. The detection and counting of Hz containing leukocytes in fresh human or mouse blood, as well as in leukocytes from *in-vitro *experiments was rapid and easy. Analysis of CD14/CD16 and CD11b/Gr1 monocyte expression in human or mouse blood, in a mixed populations of Hz-containing and non-containing monocytes, appears to show distinct patterns in both types of cells. Hz-containing pRBC and different maturation stages could be detected in blood from infected mice. The analysis of a highly depolarizing population that contained mature pRBC allowed to assess the effect of chloroquine and quinine after only 2 and 4 hours, respectively.

**Conclusions:**

A simple modification of a flow cytometer allows for rapid and reliable detection and quantification of Hz-containing leukocytes and the analysis of differential surface marker expression in the same sample of Hz-containing *versus *non-Hz-containing leukocytes. Importantly, it distinguishes different maturation stages of parasitized RBC and may be the basis of a rapid no-added-reagent drug sensitivity assay.

## Background

The malaria pigment, or haemozoin (Hz), is gaining increasing attention, as has been reviewed recently [1,2]: (i) Hz production is an important drug target, (ii) Hz appears to have immunomodulatory properties, (iii) detection of Hz-containing leukocytes allows diagnosis of malaria, and (iv) Hz-containing leukocytes appear to be associated with disease severity. However, one important drawback in this area of research is the fact that counting of Hz-containing leukocytes or the detection of Hz-containing parasitized red-blood cells (pRBCs) is based on microscopy [3,4]. This is not only cumbersome, but introduces a significant statistical error if the number of Hz-containing leukocytes is low [5]. An alternative to this is based on the detection of depolarized Side-Scatter by flow cytometry [6]. Hz is birefringent and as a consequence rotates the plane of polarized light, a process called depolarization. LASER light, commonly used in flow cytometers, produces polarized light. Thus, by placing a polarization filter orthogonally (90° rotated) to the plane of the LASER light in front of a second SSC detector allows to detect depolarized light and consequently Hz.

Depolarized SSC detection is incorporated into the Cell-Dyn^® ^haematology analysers (Abbott, Santa Clara, CA, USA) to differentiate eosinophils from granulocytes. As a result of this, these instruments detect Hz-containing leukocytes as well as Hz in parasitized red blood cells (pRBC) without need for modifications, [7,8]. Unfortunately, the software analysis and analysis algorithms of the analysers cannot be accessed without the intervention of the manufacturer and thus, studies depended on counting the events (cells) on the instrument's screen or the printout, with consequent data loss and no option for further analysis of the raw data [9]. However, if a simple modification of common flow cytometers created a reliable method for detection and analysis of Hz in leukocytes or pRBC it might open novel approaches for diagnostic applications and research.

For example, flow cytometric counting of Hz-containing leukocytes may be a better marker for disease severity than microscopy based counts [5,10]. Detection and functional analysis of Hz-containing leukocytes may also help to elucidate further aspects on the immunomodulatory properties of Hz. In fact, most *in-vitro *studies use concentrations of Hz that maximize uptake by the majority of monocytes in a given cell population and compare them with a control population that was not exposed to Hz [11]. However, the situation may be different *in vivo*, where both populations coexist. For example, a recent large study, based on microscopy, reported that the median percentage of circulating Hz-containing monocytes was only 2-5% [3].

Interestingly, monocytes are a heterogeneous cell population, even in peripheral blood [12,13], mainly based on the CD14 and CD16 expression: classical "inflammatory" monocytes (CD14^+^/CD16^-^), "intermediate" monocytes (CD14^+^/CD16^+^) and "resident, pro-inflammatory" monocytes (CD14^dim^/CD16^+^). They also seem to differ functionally [14]. Recent research showed differences in the ratio of these populations in malarious children with different types of malaria [15], as well as differences in surface marker expression and parasite inhibitory action in acute uncomplicated malaria [16]. However, little is known whether Hz phagocytosis contributes to these findings *in vivo*. In fact, the interplay of Hz-containing and non-Hz-containing subpopulations might produce different biological results *in vivo*, when compared to those *in vitro *models, where nearly all monocytes contain Hz.

Another important area for flow cytometric detection of Hz could be the reliable detection of pRBC harbouring different Hz content. This may be useful for sensitivity testing and drug development, because the Hz-content increases proportional to the maturation of the parasite [17].

This paper describes a simple way to modify a standard bench-top flow cytometer to allow depolarized Side-Scatter measurements. The method was then tested for its usefulness to detect Hz-containing leukocytes and erythrocytes and examples for potential applications are presented.

## Methods

### Flow cytometer modification (depolarized Side Scatter detection)

**Figure 1:**
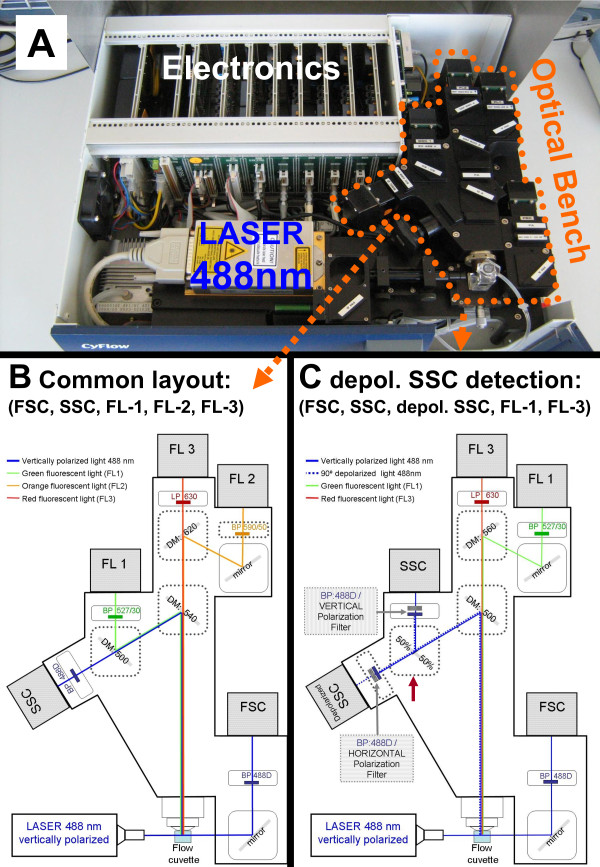
**Alterations to the optical bench of a common bench-top flow cytometer which allows detection of depolarized side-scatter**. a) The lid of the CyFLow^® ^Blue flow cytometer can be easily removed and filters can be swapped by the operator. b) Light path in conventional filter set-up for detection of Forward Scatter (FSC), Side-Scatter (SSC), green (FL-1), orange (FL-2) and red (FL-3) fluorescence. c) Filter set-up that allows detection of depolarized Side-Scatter instead of FL-2 detection. Squares with broken line indicate dichroic mirrors that need to be changed. Red error shows 50%/50% beam splitter. Other beam splitters which divert more light to the depolarized SSC are possible, such as 90%/10% or even 95%/5%. DM = Dichroic Mirror; BP = Bandpass filter, LP = Longpass filter, numbers indicate wavelength in nm.

The CyFlow^® ^Blue (Partec, Münster, Germany) is a small, five parameter flow cytometer (Figure 1a) with blue laser (488 nm) excitation, and detectors for Forward Scatter (FSC), Side Scatter (SSC), green fluorescence (FL1), orange fluorescence (FL2) and red fluorescence (FL3) (Figure 1b). For this study the set-up was changed, mainly by creating two SSC detectors, using a 50%/50% beam splitter. Then a polarization filter was placed orthogonally to the polarization plane of the laser light, in front of one of these SSC detectors which allowed the detection of depolarized Side-Scatter. This and other necessary changes are indicated in Figure 1c.

### Reagents

All reagents were obtained from Sigma Aldrich (St Louis, Mo, USA), unless stated otherwise. Fluorescein isothiocyanate (FITC) or phycoerythrin (PE) labelled antibodies against surface antigens as well as isotype antibodies (CD14-FITC, CD16-PE, CD11b-FITC, Gr1-PE, TER119-PE, Fc-block) were purchased from eBioscience (San Diego, USA). DNAse/RNAse free ultrapure water, Phosphate buffer saline (PBS), Fetal Bovine Serum (FBS), HEPES and RPMI 1640 were purchased from Gibco (Grand Island, NY). Ultrapure water was obtained with a Milli-Q purification system (Millipore, Madrid, Spain).

### Haemozoin preparation and quantification

Synthetic haemozoin (sHz) was prepared by dissolving 1.1 gr of hemin chloride in 70 ml of 0.4 M NaOH and 62 ml of ultrapure water. The heme was then precipitated by the addition of 68 ml of glacial acetic acid. The mixture was heated at 37°C over night to promote the formation of β-haematin. The following day, the formed crystalline synthetic haemozoin (sHz) was washed 5 times in 5% pyridine (in 0.02 M HEPES at pH 7.5) and five times in ultrapure water. Finally, sHz was resuspended in PBS, quantified and stored at 4°C.

*Plasmodium. falciparum *(strain 3D7) haemozoin (Pf-Hz) was purified from a synchronized culture at a parasitaemia of around 3-5%. Infected erythrocytes were spun at 2000 rpm for 10 min and resuspended for 10 min in a mixture of 40 ml ultrapure water with 2 ml of a 1% saponin in water. Cell lysates were then centrifuged at 13,200 rpm for 15 min. The Hz pellets were washed 4x with PBS, resuspended in 1 ml PBS, quantified and stored at 4°C. Both types of Hz showed no DNA contamination and were negative for *Mycoplasma*.

The concentration of Hz was determined as haem content (haem-equivalent) after solubilization in 20 mM NaOH for 1 hour at room temperature. The haem concentration was then determined by luminescence at 400 nm using the QuantiChrom Heme Assay Kit from BioAssay Systems (Hayward CA, USA).

### In vitro incubation of human whole blood with synthetic haemozoin

Heparin-anticoagulated blood from healthy human donors was diluted 1:1 in RPMI 1640; and distributed into a 24 well plate. Then sHz was added at 0.01; 0.06 and 0.12 μmol haem-equivalent/ml. The plate was incubated for 7 hours at 37°C in 5% CO2. Leucocytes were analysed in triplicates at time 0, 4 and 7 hours.

### In vitro incubation of human PBMCs with synthetic and *P. falciparum *haemozoin

Human PBMC were isolated from 40 ml heparin-anticoagulated blood collected from healthy volunteers and after dilution 1:1 in RPMI 1640 and placed in a Ficoll gradient (Ficoll-Paque Plus, GE Healthcare, Uppsala, Sweden). Cells were centrifuged at 700 g for 20 min and the interface containing the PBMCs was collected. The PBMCs were washed, counted and resuspended at a concentration of 1 × 10^6 ^PBMC/ml in RPMI 1640 supplemented with 2 mM L-glutamine, 0.05 mg/ml gentamicin and 10% foetal calf serum and distributed into a 24 well plate. Then sHz was added at 0.004 and 0.007 μmoles heme-equivalent/ml and P.f.-Hz was added at 0.002 and 0.004 μmol haem-equivalent/ml in triplicates. Polystyrene latex beads (0,1 μm) were diluted from the stock at 10% (vol/vol) to a final concentration of 0.001% and used as control. The plate was incubated for 6 hours at 37°C in 5% CO2.

### Flow cytometric analysis of human leukocytes

EDTA anticoagulated blood was obtained from volunteers and patients for immediate analysis. One hundred μl of EDTA-anticoagulated blood was incubated for 20 minutes with anti-CD14 and anti-CD16. RBC were then lysed prior to analysis with Whole Blood Lysis Reagent (Partec, Münster, Germany) without further washing.

**Figure 2:**
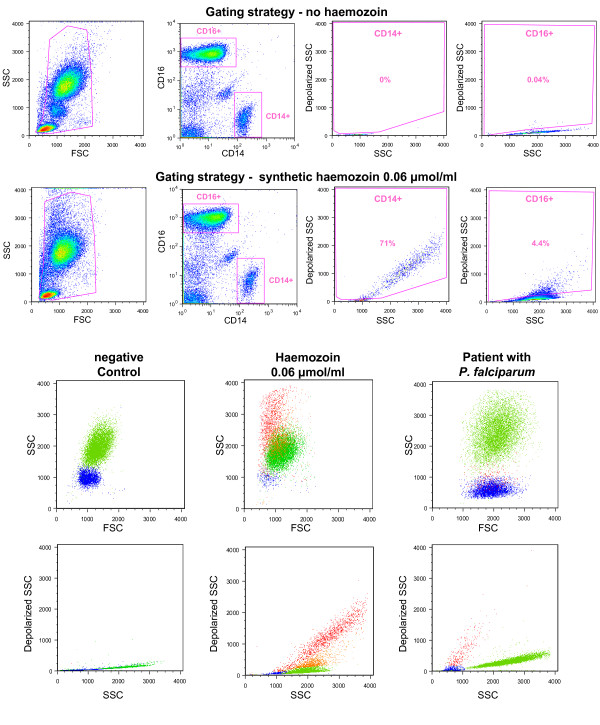
**Detection of Hz-containing monocytes and granulocytes in human whole blood**. Top rows: Gating strategy for detection of haemozoin (Hz) containing monocytes (CD14+) and granulocytes (CD16+). Top row: blood from healthy donor without Hz, second row: incubated for 7 hours with synthetic haemozoin (0.06 μmol/ml heme-equivalent). Using plots from healthy volunteers (top row) gates were created to identify depolarizing monocytes and granulocytes. Bottom two rows: Colour backgating for samples shown in row one and two, as well as for a patient with *P. falciparum *malaria (0.2% parasitaemia). Blue = non-depolarizing monocytes, red = depolarizing monocytes, green = non-depolarizing granulocytes, orange = depolarizing granulocytes.

For the *in vitro *whole blood assay 50 μl were incubated with anti-CD14 and anti-CD16 antibodies. RBC were lysed with BD FACS lysing solution (BD Biosciences, San Jose) for 5 minutes and washed before analysis. PBMCs were labelled as described for the whole blood assay, without erythrocyte lysis. All *in-vitro *samples were performed in triplicate. The gating strategy for monocytes and granulocytes and identification of Hz-containing leukocytes is shown in Figure 2.

### Flow cytometric analysis of murine leukocytes

Groups of five C57BL/6 mice (Charles River, Spain) were infected intraperitoneally with *Plasmodium berghei *ANKA (PbA) as model for experimental cerebral malaria or *P. berghei *NK65 (PbNK) as model for hyperparasitaemia. Uninfected and *P. berghei *NK65 infected mice were followed for 18 days. PbA-infected mice were sacrificed on day 5 when showing obvious signs of morbidity. Parasitaemia and flow cytometric analysis of blood was performed on days 3, 5, 12 and 18 post infection. At each time point approximately 25 μl of blood was collected from a tail vein, incubated with Fc-block and then labelled with anti-CD11b and anti-Gr1. After washing the cells, RBC were lysed with 125 μl of BD FACS lysing solution (BD Biosciences, San Jose) for 5 min. The cells were washed again, resuspended in FACS buffer and analysed by flow cytometry. Granulocytes and monocytes were analysed as described previously [18], in particular following a protocol that uses Side Scatter, CD11b and Gr1 expression [19] (see: additional file 1 - mouse blood gating strategy.pdf).

### Analysis of murine Hz-containing RBC

Mice (C57BL/6) (Charles River, Spain) were infected with *P. berghei *ANKA. Blood samples were collected when mice had a parasitaemia of approximately 5%. RBC were incubated with Fc-block and then labelled with anti-TER119. After washing the cells, SYBR green I (Invitrogen, Carlsbad, USA) was used to stain the DNA of intraerythrocytic parasites. Briefly, 2.5 μl of the SYBR Green I solution at 10x was added to a blood suspension of approximately 400,000 cells/mL and incubated for 20 minutes, in the dark.

### Anti-malarial drug effect on *P. berghei *infected RBC in-vitro

Blood from infected mice was diluted 1:50 in RPMI medium supplemented with 10% foetal calf serum, 1% non essential amino acids, 1% penicillin/streptomycin, 1% glutamine and 10 mM Hepes, pH 7. The blood suspension was diluted 1:1 in complete RPMI medium and incubated with either chloroquine or quinine for 12 hours in a 24 well plate at 37°C in a 5% CO2 atmosphere. Chloroquine diphosphate salt or quinine hydrochloride were dissolved in ultrapure water to prepare the stock solution. Final concentrations of 25 nM, 50 nM and 100 nM of chloroquine or 400 nM and 800 nM of quinine were used. Flow cytometric measurements were done during every two hours by diluting 5 μl of the suspension present in the wells in 1 mL of FACS buffer. The effect of the anti-malarial drugs on the parasites was assessed by quantifying the percentage of highly depolarizing events (hdRBC).

All flow cytometry results were analysed using FlowJo software (version 9.0.2, Tree Star Inc., Oregon, USA).

The study was approved by the Ethical Committee of the Faculty of Medicine, University of Lisbon. All experiments involving animals were performed in compliance with the relevant laws and institutional guidelines.

## Results

The Cyflow^® ^instrument can be easily adapted to detect depolarized Side-Scatter (depol-SSC) by the operator, without any sophisticated tools or special knowledge. It is only necessary to open the instrument's cover, after releasing four screws, and change the respective filters and dichroic mirrors (Figure 1). This is a very simple procedure that lasts approximately 5 minutes. No special software is necessary and the same set-up (linear) as used for conventional SSC detection is sufficient, although the gain (voltage) of the detectors had to be adjusted.

### Detection of Hz-containing leukocytes in human blood

**Figure 3:**
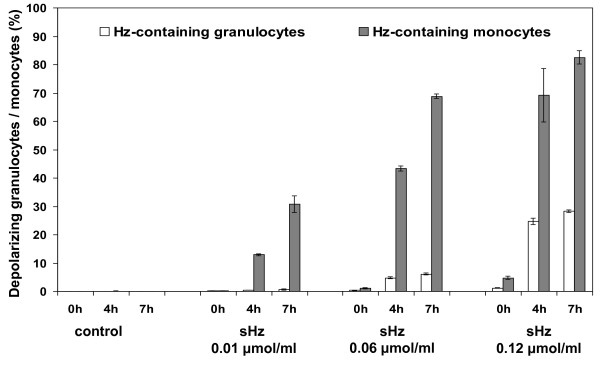
**Phagocytosis of haemozoin *in-vitro *depends on dose and incubation time**. Whole Blood from a healthy human volunteer was incubated with synthetic haemozoin (sHz) at 0.01, 0.06 and 0.12 μmol/ml of heme equivalent and with no sHz (control). The percentage of Hz-containing granulocytes (open bars) and monocytes (grey bars) was determined at time zero, and after four and seven hours of incubation. Identification of leukocytes and depolarization as described in the text and shown in figure 2. Results are the mean values of triplicates (± one SD)

Using anti-CD14 and anti-CD16 it was possible to identify Hz-containing monocytes and granulocytes in whole blood from healthy human volunteers incubated with Hz (Figure 2 and 3) as well as in a sample from a patient with malaria (Figure 2). Importantly, Hz-containing monocytes show much higher Side-Scatter than normal monocytes, which overlap with the granulocyte population (Figure 2, bottom rows).

Incubation of whole blood from healthy volunteers with sHz shows that the percentage of Hz-containing monocytes and granulocytes depends on the initial Hz-dose and time of incubation (Figure 3).

**Figure 4:**
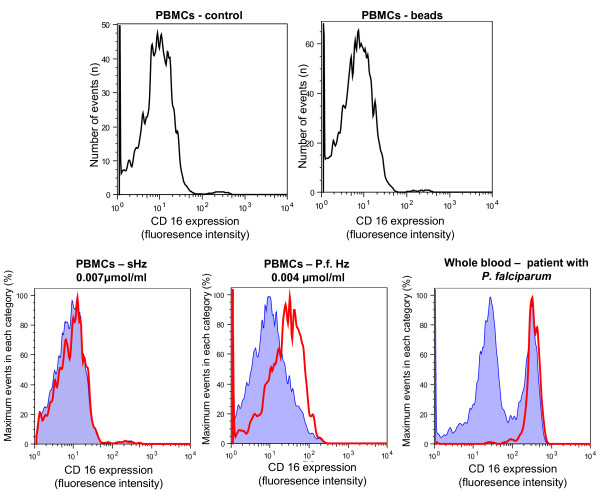
**Different expression of CD16 on Hz-containing monocytes**. PBMCs were isolated from blood of a healthy human volunteer and were incubated with synthetic Hz (0.007 μmol/ml heme-equivalent) and with *Plasmodium falciparum *Hz (P.f.Hz) at 0.004 μmol/ml heme-equivalent. PBMCs incubated with latex beads and with no Hz were used as controls. CD14+ monocytes were gated, analysed for depolarization and then analysed for CD16 expression. Patient with *P. falciparum *malaria (0.2% parasitaemia) 2 days into treatment (bottom right panel). Blue shaded area: non-depolarizing monocytes, red line depolarizing monocytes.

**Figure 5:**
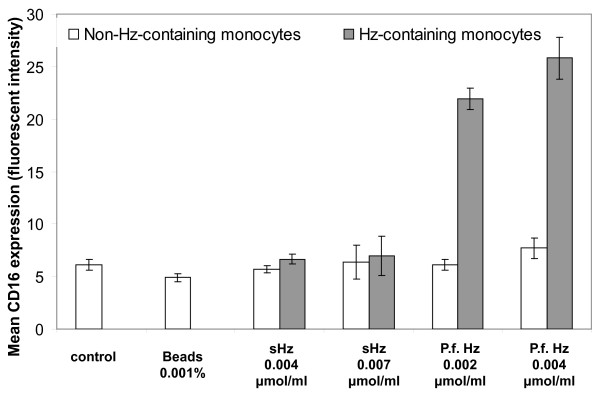
**CD16 expression on monocytes fed with different types of haemozoin *in-vitro***. PBMCs were isolated from blood of a healthy human volunteer and were incubated with synthetic Hz (sHz) at 0.004 and 0.007 μmoles/ml heme equivalent and with *Plasmodium falciparum *Hz (P.f.Hz) at 0.002 and 0.004 μmol/ml heme equivalent. PBMCs incubated with latex beads and with no Hz were used as controls. Cells were incubated for 6 hours and then labelled with anti-CD14 and anti-CD16. The Geometric mean values for expression of CD16 were calculated for non-Hz containing monocytes (open bars) and Hz-containing monocytes (grey bars). Differences between sHZ and P.f. were significant (P < 0.01). Shown are values of triplicates (± one SD). Controls and beads had no depolarizing populations.

In addition, different types of Hz cause distinct expression of CD16 on Hz-containing monocytes, as compared with non-haemozoin containing monocytes in mixed populations (Figure 4, 5). Contrary to the synthetic Hz used in this study, *P. falciparum *derived Hz increases significantly CD16 expression on Hz-containing monocytes from healthy volunteers (*P *< 0.01); Figure 4 and 5). Interestingly, in a single case of *P. falciparum *malaria with 5.4% Hz-containing monocytes, a substantial increase of CD16 expression was noticed and the majority of HZ-containing monocytes were clustered in the CD14+/CD16+ subpopulation (Figure 4, bottom row, right).

Altogether, the data clearly show that a simple alteration in a bench-top flow cytometer for detection of depolarized side-scatter allows quantification of Hz-containing monocytes, which could be easily detected in a case of *P. falciparum *malaria.

### Hz-containing murine leukocytes

**Figure 6:**
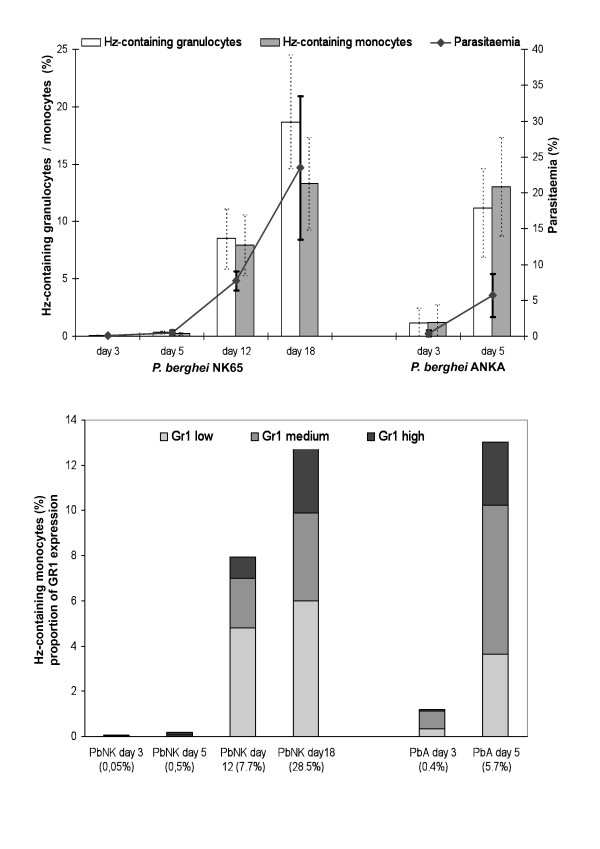
**Haemozoin containing leukocytes in two different mouse models of malaria**. Groups with five C57BL/6 mice where infected with *P. berghei *ANKA (PbA) or with *P. berghei *NK65 (PbNK). Blood was drawn on day 3, 5 12 and 18 and analysed after labelling with with anti-CD11b and Gr1. Analyses and gating strategy is described in the text (additional file 1). Percentage of Hz-containing monocytes and granulocytes (top graph) as well as parasitaemia are mean values per group (± one SD). Open bars = Hz-containing granulocytes; grey bars = Hz-containing monocytes. Uninfected controls had no Hz-containing leukocytes and are not shown. Bottom graph shows proportion of different Gr1 expression (high = black, medium = grey; and low = light grey) of Hz-containing monocytes, shown in top figure. Percentages on x-axis represent mean parasitemia. Mice with PbANKA infection were sacrificed on day 5.

Using anti-CD11b and anti-Gr1 it was possible to identify Hz-containing monocytes and granulocytes in mouse blood (Figure 6). Mice with PbA infection were sacrificed on day 5 because they developed symptoms compatible with experimental cerebral malaria. Overall, the results show that the percentage of Hz-containing leukocytes increased when parasitaemia was higher (Figure 6). However, when mice infected with the two different parasite strains were compared when they had a similar parasitaemia, i.e. day 5 for PbA (5.7 ± 3.1%) and day 12 for PbNK (7.7 ± 1,3%), PbA infected mice had higher percentages of Hz-containing monocytes than PbNK infected mice (Figure 6, top graph). Also, while the levels of parasitaemia of PbNK at day 18 of infection were significantly higher than the levels of parasitaemia of PbA at day 5 of infection (28.5 ± 12% and 5.7 ± 3.1%, respectively, *P *< 0,001) the percentages of Hz-containing monocytes were almost the same (13,3% and 13,0%, respectively). Furthermore, a comparison of the degree of Gr1 expression (low, medium, high) on the Hz-containing monocytes, appeared to show differences in these two groups of mice at similar parasitaemia (Figure 6, bottom graph).

Interestingly, using CD11b and F4/80 showed that it appears possible to identify Hz-containing tissue macrophages as shown in additional file 2 (Additional file 2 - mouse spleen macrophages.pdf).

Altogether, using two distinct rodent models of infection, Hz-containing monocytes and granulocytes can also be detected in this flow cytometer. Most importantly, the data suggest that the percentage and type of Hz-containing monocytes may also be a associated with disease severity.

### Detection of Hz in murine pRBCs

**Figure 7:**
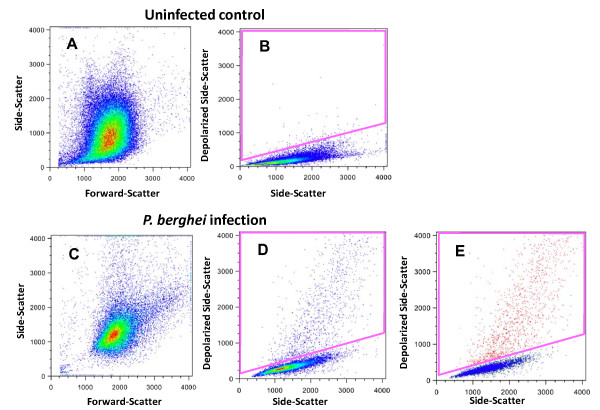
**Detection of haemozoin in *P. berghei *ANKA infected mouse RBC**. Uninfected control (upper panel). Whole blood from a C57BL/6 mouse with a parasitaemia of 8.1% (lower panel). Gate to indentify the depolarizing events in the control (0.2% events) (B) and in the infected mouse RBC (4.7% events) (D). Depolarizing events in this gate (D) were indentified as erythrocytes by anti-TER119 staining (> 97% positive) and are shown as red dots in E. Colours in plots A-D represent frequency of events. In plot E colour backgating; with blue = TER119 positive events without depolarization, red = TER119 positive events which depolarize; and green = any other event

**Figure 8:**
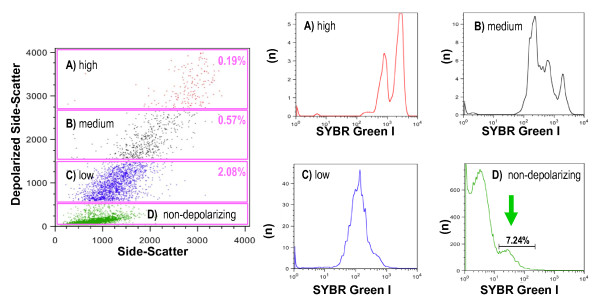
**Degree of depolarization corresponds to different developmental stages of *P. berghei *ANKA**. Blood from a C57BL/6 mouse with a parasitaemia of 9.7% as determined by microscopy. Top plot shows several populations with decreasing degree of depolarization (A, B, C). Gate D shows low or non-depolarizing events. The four histograms show the number of events (n) and their fluorescence intensity (x-axis) of SYBR Green I, representing the DNA content. Gate D contains a population of 7.24% (arrow), likely including ring-forms with little Hz and thus very low or no depolarization.

*Plasmodium *accumulates Hz throughout its development stage inside RBCs. The detection of depolarization was evaluated to determine whether this measurement could be used to detect pRBCs and most importantly to distinguish different pRBC stages. In whole blood from mice infected with *P. berghei *a population of depolarizing events is observed (Figure 7). More than 97% of these events were positive for the RBC marker TER119 (red population, Figure 7E). SYBR green I staining was positive in this population, indicating that the depolarizing erythrocytes contained DNA, and thus were parasitized RBC (pRBC) (Figure 8). Further analysis revealed that events with a higher degree of depolarization also showed a higher degree of SYBR green fluorescence (Figure 8), with several distinct peaks, likely representing different maturation stages of the parasite (ring-forms, early and late trophozoites and schizonts).

### Detection of Hz in parasitized murine RBC to assess drug effects

**Figure 9:**
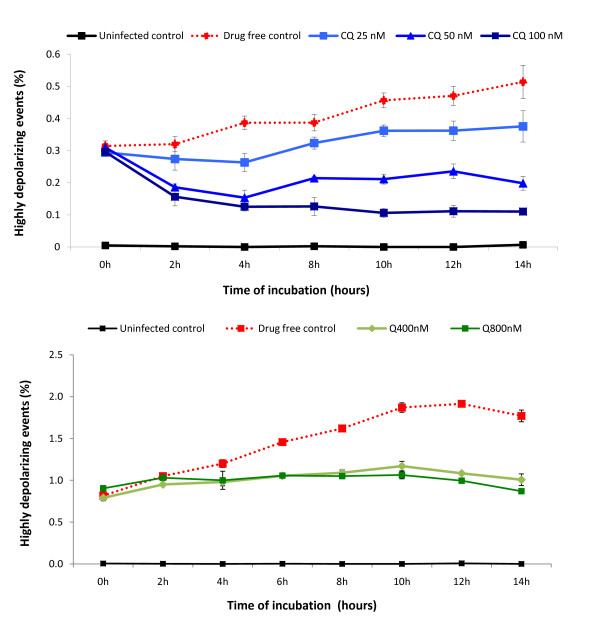
**Inhibitory effect of chloroquine and quinine on Hz containing parasites within unlysed RBC**. Blood from PbA infected C57BL/6 mice with parasitaemias around 5% were incubated with different concentrations of chloroquine (upper graph) or quinine (lower graph). The percentage of highly depolarizing events is shown (gate A in Figure 8). Each point represents the mean of triplicate measurements (± one SD). CQ = chloroquine, Q = quinine.

Based on the above findings (Figure 8, gate A), which contained the pRBC with the highest degree of depolarization (hdRBC) and as such contains the most mature parasites, the flow cytometric method was investigated to see if it could be used to rapidly assess drug effects. Thus, the percentage of this hdRBC population was determined over a 12-hour period in an *in-vitro *culture of *P. berghei *pRBC. The results clearly show that the inhibitory effect of chloroquine and quinine is detected in a dose dependent manner throughout time (Figure 9). Importantly, inhibitory effects were discernable after only two hours for chloroquine and four hours for quinine.

## Discussion

### Modification of flow cytometers

This study shows that a simple modification of a common flow cytometer for the detection of depolarized Side-Scatter, allows the detection of Hz-containing leukocytes and erythrocytes. In fact, a study using a MoFlo^® ^high speed cell sorter (Beckman Coulter, Inc, Fullerton, CA), showed that depolarizing leukocytes did contain Hz [20]. However, MoFlo^® ^sorters are expensive and sophisticated instruments, and by far not as commonly available as flow cytometers, which are even standard equipment in most research centres in malaria endemic regions nowadays. Nonetheless, the widespread incorporation of this method in common flow cytometers of major manufacturers has somehow been hampered by the fact that the company of the Cell-Dyn^® ^instruments held the patent of this method (US patent 5017497) [21]. However, this patent was filed in 1989 and published in 1991, and as such, according to US legislation [22], it expired in March 2009. The necessary filters and dichroic mirrors (Figure 1c) used to change the CyFlow^® ^cytometer were obtained for less than €2.000. No special software is necessary and the settings resemble those of the conventional SSC detection, albeit with an increased gain (voltage) of the detector. For the set-up in this study a 50%/50% beam splitter used to divert the respective light to the depolarized SSC and SSC detector (Figure 1, red error). Using a 90%/10% or even 95%/5% beam splitter may increase the signal of the depolarized SCC, without decreasing the quality of the SSC signal.

Any flow cytometer can be modified as described here. However, it should be noted that to modify some flow cytometers, which use fibre optic cables for light collection, for example, the newer Becton Dickinson instruments (Franklin Lakes, NJ, USA), it is necessary to confirm that these cables maintain polarization of the light.

### Detection and counting of Hz-containing leukocytes

Hz-containing monocytes and granulocytes could be rapidly determined in fresh human or mouse blood as well as in *in-vitro *phagocytosis experiments involving whole blood or PBMCs. As has been pointed out before, previous studies on the immunological effects of Hz *in-vitro *used diverse quantities of Hz, different incubation times, as well as different types of phagocytic cells [1,2]. Few studies quantified the uptake of Hz by monocytes, although some used a luminescence assay after lysing the monocytes and reported Hz uptake as Hz-equivalents found in around 8-10 trophozoites per monocyte [23]. However, even less studies mention the percentage of Hz-containing monocytes. In one of these studies confocal microscopy was used and showed that 75% (± 31%) of human PBMCs contained Hz after only 180 min, albeit only based on counting a total of ≥ 400 cells [11]. Contrary to this, the presented method allows the analysis of ten-thousands of cells to determine rapidly adequate doses of Hz and required incubation times to get the desired percentage of Hz containing leukocytes (Figure 3).

Furthermore, it allows to investigate Hz-containing monocytes from malarious patients who usually have only a few percent of these cells in circulation [3]. For example, considering that in common protocols for the analysis of whole blood, 100 μl are labelled, and further assuming 1,000 monocytes/μl with only 5% of these containing Hz, a total of 5000 Hz-containing cells could be easily analysed using the flow cytometric method proposed here. As previously reported [5,10], this method also allows to re-address the question if the number of Hz-containing leukocytes is associated with disease severity or certain types of malaria.

In some studies, however, the identification of monocytes was based on the typical monocyte gate in the Forward Scatter (FSC)/SideScatter (SSC) plot [16,20]. One problem with this is that monocytes containing several or larger Hz crystals might thus be excluded from analysis as they have higher SSC and appear superimposed on the granulocyte population (Figure 2), with a possible impact on final results.

### Single cell analysis for studies of Hz-containing leukocytes

This may open the possibility to investigate functional differences of Hz-containing and non-containing leukocytes, especially in monocyte subsets found in fresh human blood from malarious patients or in mouse models of malaria. For example, recent papers reported differences in surface markers on CD14/CD16 monocyte subsets in malarious patients [15,16], or in peripheral blood dendritic cells in children [24]. Measurement of depolarized Side-Scatter and, thus, identification of Hz-containing cells might allow to establish if these cells may have an imported role in the reported results. It would also allow the possibility to study functionally leukocytes on single cell level *in-vitro*, including surface expression or intracellular cytokines, comparing Hz with non-Hz containing leukocytes in the same cell population. In fact, differences in CD16 expression in a mixed population of Hz-containing and non-containing cells were observed (Figures 4 and 5), a finding that differs from a previous report that found no difference in CD16 expression in an *in-vitro *model [25]. Despite recent reports of differences between human and mouse monocytes [26], the current method may also allow to investigate the effects of Hz on phagocytic cells in fresh blood using different mouse models (Figure 6).

First results (additional file 2) indicate that it appears possible to detect tissue macrophages. This may allow to compare Hz-containing and non-Hz-containing cells from tissues such as spleen, liver or bone marrow which may help to elucidate recent findings, for example, the association of Hz with severe anaemia [27,28]. However, the reliable identification of tissue macrophages may require more than the two antibodies used in this study. In this case, instruments that have several light sources are necessary.

### Detection of pRBC and sensitivity testing of antimalarious drugs

Flow cytometric analysis of depolarized Side-Scatter allows to easily detect pRBCs (Figure 7) and distinguish different maturation stages of the parasite (Figure 8). Both findings might serve as the basis for a novel drug sensitivity assay. In fact, the idea to use Hz as a maturation indicator is not new and was tried in a simple visual agglutination test in the 1980s [17]. Apparently, this method did not produce very reliable results, mainly due to leukocyte interference [29]. However, these limitations could be overcome by single-cell flow cytometric analysis.

Currently several drug sensitivity assays for *P. falciparum *exist and have been reviewed elsewhere [29,30]. Nonetheless, all of these assays have some disadvantages, including long turn-around times or the need for additional and often expensive reagents. Interestingly, a recent report concluded that the reliability of some of these assays may depend on the mode of action of the tested drugs [31]. Flow cytometry appears to be a reliable technique to assess parasite maturation and sensitivity to antimalarial drugs. However, protocols reported so far require additional reagents, usually to stain DNA and/or RNA and may even require flow cytometers with more than one light source [32,33].

The presented method is based on the detection of Hz, without the need of any further reagents. Determining the percentage of highly depolarizing RBC (hdRBC) present in blood samples from infected mice, it was possible to detect the inhibitory effect of chloroquine (CQ) and quinine (Q) after only a few hours of *in-vitro *culture (Figure 9). In this study an experimental model using C57BL/6 mice infected with *P. berghei *was chosen to assure that only maturation of parasites was measured, because *P. berghei *does not replicate in *in-vitro *cultures [34]. However, although the preliminary results seem promising, further work with different drugs and, most importantly, *P. falciparum *cultures are necessary to evaluate the usefulness of this method as a novel sensitivity assay.

## Conclusions

A simple modification of a flow cytometer allows the reliable detection of Hz containing leukocytes and pRBCs. This method facilitates the rapid and reliable detection and counting of Hz-containing leukocytes in human or animal blood. It also allows single cell analysis of Hz-containing *versus *non-Hz-containing leukocytes in the same sample, including functional analysis, both *in-vitro *experiments or using fresh blood. Finally, it detects different maturation stages of parasitized RBC and may be the basis of a rapid no-added-reagent drug sensitivity assay.

## Competing interests

The authors declare that they have no competing interests.

## Authors' contributions

RF and MR contributed equally to this paper.

RF carried out the human and mouse leukocyte studies. MR realized the studies on parasitized RBC and anti-malarious drug effects. AP, AMV and MMM participated in setting up the mouse models and analysing the results. MMM and TH conceived the study, participated in its design and coordination. MPG contributed to the design of the study and the draft of the manuscript. All authors have read the manuscript and approved it.

## Supplementary Material

Additional file 1 Description of flow cytometric gating strategy to indentify Hz-containing mouse granulocytes and monocytes. Click here for file

Additional file 2 Detection of Hz-containing mouse spleen macrophages. Click here for file
