# Supplementary figures and images for: Simple flow cytometric detection of haemozoin containing leukocytes and erythrocytes for research on diagnosis, immunology and drug sensitivity testing
Source: Malar J. 2011 Mar 31;10:74. doi: 10.1186/1475-2875-10-74 (PMC3078904; doi:10.1186/1475-2875-10-74)

## Uninfected mouse (negative Control)

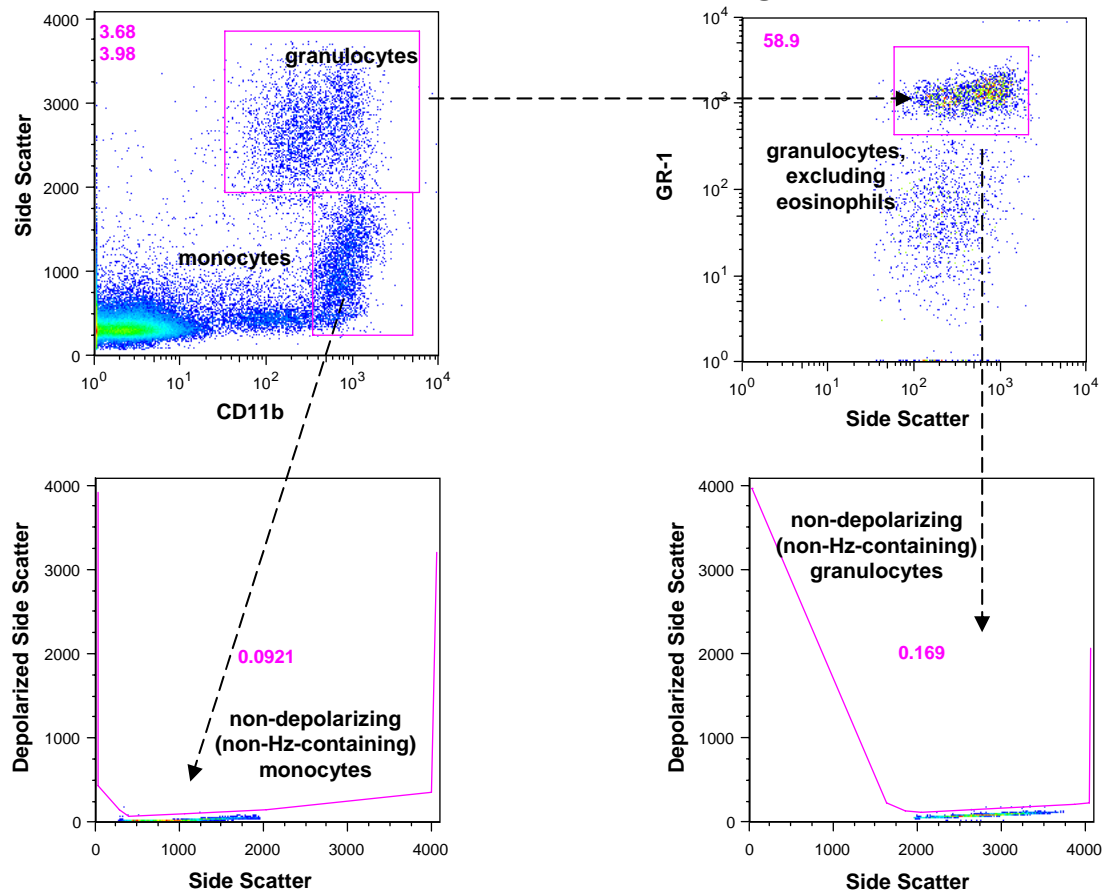

## PbNK infected mouse (day 12)

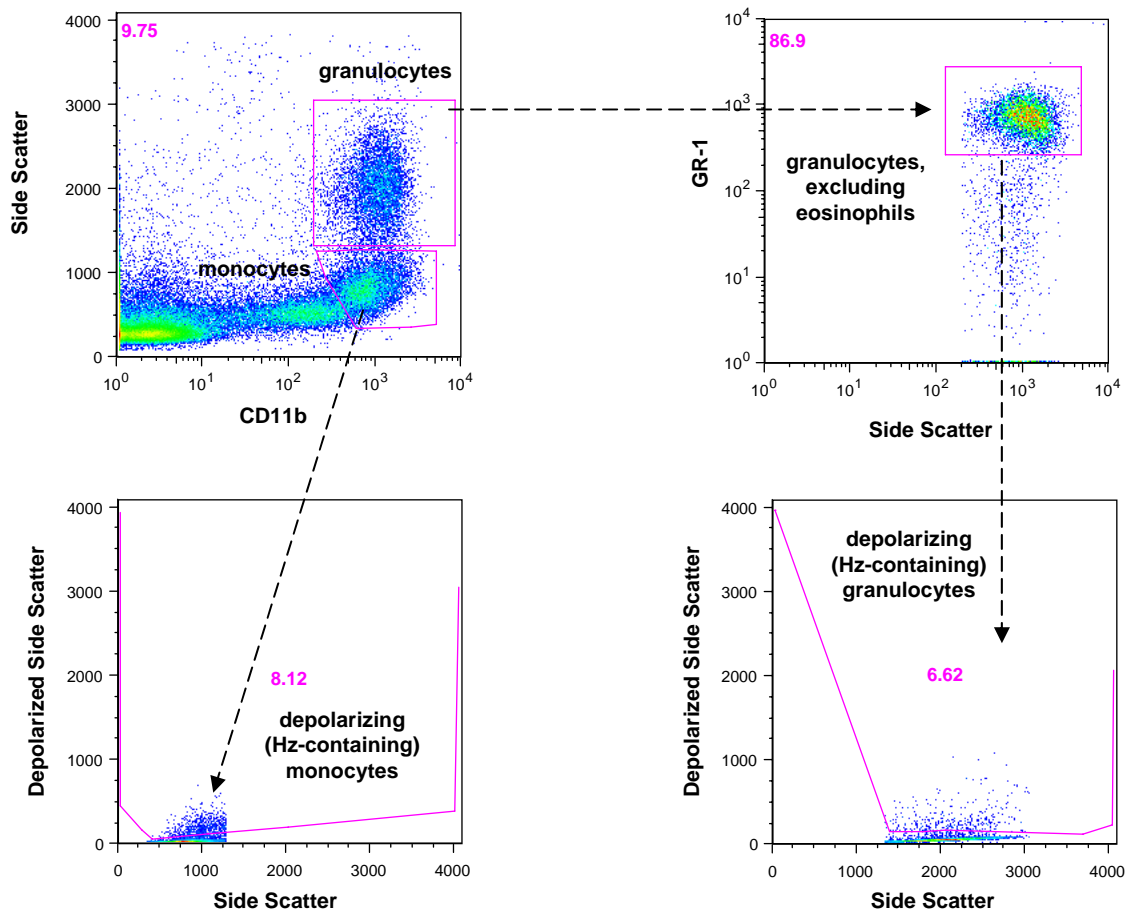

Supplement: Additional file 1 — Description of flow cytometric gating strategy to indentify Hz-containing mouse granulocytes and monocytes. [file 1475-2875-10-74-S1.PDF]
