# Supplementary material for: Simple flow cytometric detection of haemozoin containing leukocytes and erythrocytes for research on diagnosis, immunology and drug sensitivity testing
Source: Malar J. 2011 Mar 31;10:74. doi: 10.1186/1475-2875-10-74 (PMC3078904; doi:10.1186/1475-2875-10-74)
Supplement: Additional file 2 — Detection of Hz-containing mouse spleen macrophages. [file 1475-2875-10-74-S2.PDF]

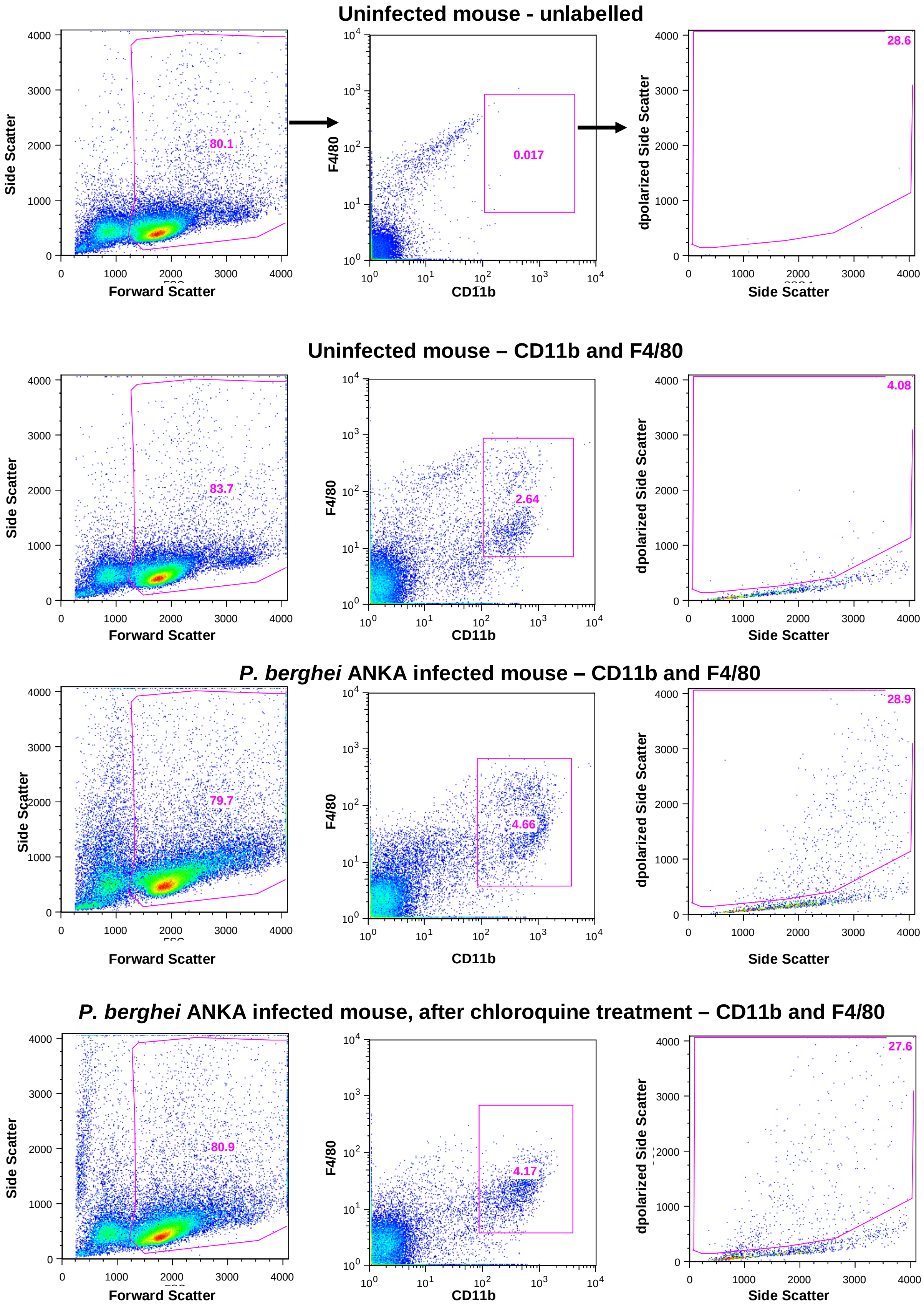

Additional file 2 - mouse spleen macrophages:

Spleens were aseptically removed from naïve, infected (10% pRBC) and infected chloroquine-treated (0.5% pRBC) C57BL/6 perfused mice. A single-cell suspension was obtained by mincing off the spleen and passing the disrupted tissue through a 70-µm pore-size nylon strainer (BD Falcon). After washing and centrifugation at 500*g* for 10 minutes, cell pellets were resuspended for 5 minutes in 8.3 g/LNH4Cl in 0.01M Tris-HCl pH 7.5 to lyse RBCs. Spleen cells were washed and the numbers of cells per spleen were counted using a Neubauer chamber. Spleen cells (10^6) were incubated with FcR block and labelled with CD11b (FITC) and F4/80 (PE). Some 50.000 events were acquired. Gating strategy: a) gating on cell population in Forward-Scatter/Side-Scatter (left column), b) identification of cell-population, double positive for CD11b and F4/80 (middle column), c) determination of depolarizing (Hz-containing) cells. Numbers are percentages of parent gate.
